# Synthetic Peptide Libraries Designed From a Minimal Alpha-Helical Domain of AS-48-Bacteriocin Homologs Exhibit Potent Antibacterial Activity

**DOI:** 10.3389/fmicb.2020.589666

**Published:** 2020-11-12

**Authors:** Jessica N. Ross, Francisco R. Fields, Veronica R. Kalwajtys, Alejandro J. Gonzalez, Samantha O’Connor, Angela Zhang, Thomas E. Moran, Daniel E. Hammers, Katelyn E. Carothers, Shaun W. Lee

**Affiliations:** ^1^Department of Biological Sciences, University of Notre Dame, Notre Dame, IN, United States; ^2^Eck Institute for Global Health, University of Notre Dame, Notre Dame, IN, United States; ^3^Department of Chemistry and Biochemistry, University of Notre Dame, Notre Dame, IN, United States

**Keywords:** bacteriocin, antimicrobial peptide, enterocin, synthetic peptide, antibiotics

## Abstract

The circularized bacteriocin enterocin AS-48 produced by *Enterococcus* sp. exhibits antibacterial activity through membrane disruption. The membrane-penetrating activity of enterocin AS-48 has been attributed to a specific alpha-helical region on the circular peptide. Truncated, linearized forms containing these domains have been shown to preserve limited bactericidal activity. We utilized the amino acid sequence of the active helical domain of enterocin AS-48 to perform a homology-based search of similar sequences in other bacterial genomes. We identified similar domains in three previously uncharacterized AS-48-like bacteriocin genes in *Clostridium sordellii*, *Paenibacillus larvae*, and *Bacillus xiamenensis*. Enterocin AS-48 and homologs from these bacterial species were used as scaffolds for the design of a minimal peptide library based on the active helical domain of each bacteriocin sequence. 95 synthetic peptide variants of each scaffold peptide, designated *Syn-enterocin*, *Syn-sordellicin*, *Syn-larvacin*, and *Syn-xiamensin*, were designed and synthesized from each scaffold sequence based on defined biophysical parameters. A total of 384 total peptides were assessed for antibacterial activity against Gram-negative and Gram-positive bacteria. Minimal Inhibitory Concentrations (MICs) as low as 15.6 nM could be observed for the most potent peptide candidate tested, with no significant cytotoxicity to eukaryotic cells. Our work demonstrates for the first time a general workflow of using minimal domains of natural bacteriocin sequences as scaffolds to design and rapidly synthesize a library of bacteriocin-based antimicrobial peptide variants for evaluation.

## Introduction

New chemical and biological scaffolds for the design of antibiotic compounds have become a highly prioritized area of research, especially as novel pandemics and secondary bacterial infections become a wider global concern (Rosenberg and Goldblum, 2006). Antimicrobial peptides (AMPs) represent a broad source of chemical and functional discovery for the development of novel antibiotics (Boman, 2003). Although AMPs are highly diverse, and span the range from prokaryotes to lower and higher eukaryotes, many AMPs have conserved structural features, with common amphiphilic and alpha helical domains (Jenssen et al., 2006; Fjell et al., 2012; Uggerhøj et al., 2015; Haney et al., 2017). A common mechanism by which AMPs exert direct antimicrobial activity involves the accumulation of the peptide on the bacterial cell surface, with subsequent membrane disruption, leading to cell death (Nakajima, 2003; Brogden, 2005; Lv et al., 2014; Ong et al., 2014; Mahlapuu et al., 2016). Strategies to improve the activity of natural AMPs have focused on biophysical approaches to increase both the targeting affinity for anionic bacterial membranes and to improve their ability to penetrate lipid membrane domains, as well as altering properties including heat stability, solubility, and protease resistance for more effective therapeutic use (Fjell et al., 2012; Field et al., 2015a; Mikut et al., 2016; Torres et al., 2019).

The AMPs of bacteria, known broadly as bacteriocins, are a group of genetically encoded and ribosomally produced peptides that exist in operons containing the genes necessary for its assembly and export (Alvarez-Sieiro et al., 2016). Although bacteriocins are highly diverse in structure and function, the most fundamental division of bacteriocins, based on structure, is into class I (modified) and class II (unmodified) types (Cotter et al., 2013; Alvarez-Sieiro et al., 2016). In both cases, bacteriocins are believed to undergo the cleavage of a leader sequence from the core peptide domain. In addition to leader sequence cleavage, class I peptides are subject to additional post-translational modifications, including heterocyclization, glycosylation, and head-to-tail circularization (Arnison et al., 2013; Cotter et al., 2013; Alvarez-Sieiro et al., 2016). The class I bacteriocin nisin, which has been widely approved for use as a food preservative, is currently being researched for increased activity against infectious bacteria (Field et al., 2015a, c, 2019). Nisin is distinguished by post-translational installation of dehydroalanine and a thioether polycyclic lanthionine bridge (Field et al., 2015b; Murinda et al., 2003). Thiopeptides, a type of class I bacteriocin containing thiazole rings, have been used for the development of antibiotic lead compounds for the treatment of *Clostridium difficile* infections. Using a naturally occurring thiopeptide as a scaffold, the compound LFF57 was designed by using traditional medicinal chemistry and structure activity relationship approaches and is now in clinical trials (Lamarche et al., 2012; Mullane et al., 2015). Despite these significant advances, however, bacteriocins are still highly underrepresented as template sources for the design of linear AMPs.

Enterocin AS-48 is a circular, largely unmodified bacteriocin (Class I or Class II) produced by *Enterococcus* sp. and has been most commonly studied as a possible food preservative (Gálvez et al., 1989; González et al., 2000; Gómez et al., 2012; Caballero Gómez et al., 2013; Burgos et al., 2015). This bacteriocin is first produced as a prepropeptide consisting of a leader sequence and a propeptide. Subsequent proteolytic cleavage of the leader sequence results in a propeptide that undergoes head to tail macrocyclization to produce the active product (Sánchez-Hidalgo et al., 2011). Mature AS-48 consists of five alpha helices, with cationic residues clustered within helices four and five. These residues have been hypothesized as critical for the antimicrobial activity of AS-48 (Jiménez et al., 2005; Montalbán-López et al., 2011; Sánchez-Hidalgo et al., 2011). However, peptide variants consisting of portions of this region obtained by limited proteolysis or chemical synthesis were not found to retain full antibacterial activity (Montalbán-López et al., 2008, 2011; Sánchez-Hidalgo et al., 2011).

Our previous studies have demonstrated that active regions of bacteriocins such as AS-48 can be minimized and utilized as scaffolds to design novel, bacteriocin-based minimal peptides (Fields et al., 2018). Using rational design criteria based on a set of biophysical parameters, minimal domain bacteriocin library variants can be designed and tested for improved antibacterial activity. Several key biophysical parameters have been shown to improve the activity of the library peptide candidates (Uggerhøj et al., 2015; Fields et al., 2018). The substitution of basic residues in the minimal peptide increases the affinity of the peptide for the bacterial membrane (Fjell et al., 2012). Increased incorporation of hydrophobic residues at specific locations can also improve the ability of the minimal peptide to penetrate the lipid membrane domain (Uggerhøj et al., 2015). Further, since AMPs are typically amphipathic, key amino acid substitutions to increase the amphipathic nature of the minimal peptide candidate can also improve the ability of the peptide to target, bind to, and disrupt the bacterial membrane (Ong et al., 2014; Wang et al., 2017). In a general heuristic for library design, these amino acid substitutions can be systematically incorporated and evaluated in assays to gain additional insights into a general strategy for improved antimicrobial peptide design.

In this study, we sought to develop a systematic library-based synthetic strategy for the design and testing of linear reductive AMPs from gene sequences of full-length AS-48 bacteriocins. We utilized the known AS-48 sequence from *Enterococcus* sp. as well as three previously unidentified AS-48-like bacteriocin genes present in *Clostridium sordellii*, *Paenibacillus larvae*, and *Bacillus xiamenensis* as scaffolds for the design of a minimal peptide library based on the active helical domain of each bacteriocin sequence. Our efforts present a systematic approach to identifying and verifying portions of AS-48 bacteriocins as scaffolds for the design of synthetic AMPs and their subsequent optimization for charge and hydrophobicity using a library-based approach.

## Materials and Methods

### Peptide Design

The sequence of the cationic, alpha helical regions four and five from enterocin AS-48 was subjected to protein BLAST to identify homologous regions in other bacterial species. This peptide domain was confirmed to be highly conserved in *C. sordellii*, *P. larvae*, and *B. xiamenensis*, confirming the identified peptides as AS-48-like bacteriocins (Supplementary Table S1). The libraries were rationally designed to increase antimicrobial activity of the four peptide scaffolds using three approaches. (1) Single amino acids, within the helical wheel, were flipped in a step-wise fashion to slowly increase the amphipathic nature of the peptide (Fjell et al., 2012). (2) A single, basic amino acid substitution was made, switching a short-chained amino acid for lysine, hypothesized to increase affinity of the peptides to an anionic bacterial membrane (Morita et al., 2013; Jin et al., 2016). (3) Aliphatic and non-polar short-chained amino acids were replaced with tryptophan, theorized to increase the ability of the peptides to penetrate bacterial membranes (Fimland et al., 2002; Nguyen et al., 2010). The libraries were subsequently named for their parent peptide scaffold as syn(synthetic)-enterocin, syn-sordellicin, syn-larvacin, and syn-xiamencin. Each library was composed of 96 peptides, including 95 variants in addition to the parent peptide.

### Peptide Synthesis

All 384 peptide sequences, which contained 25 amino acids each, were commercially synthesized by GenScript (Piscataway, NJ, United States). Synthesis was confirmed to be >95% pure and was verified by HPLC and mass spectrometry prior to use (GenScript). All peptides were dissolved in 10% DMSO to final stock concentrations of 1.28 mM. For circular dichroism (CD) spectroscopy, peptides were dissolved in nanopure water to minimize background noise.

### Computational Structure Modeling

Three-dimensional peptide models were predicted using the PEPFOLD online server and visualized using PyMOL (Maupetit et al., 2009). Helical wheel predictions, to illustrate changes in amphipathicity, were made via EMBOSS: pepwheel predictive software (Schiffer and Edmundson, 1967).

### Peptide Screening and MIC Determination

Peptides were screened against *Escherichia coli* (BL21), *Streptococcus pyogenes* (M1T1 5448, hereby referred to as M1), *Pseudomonas aeruginosa* (PAO1), and *Staphylococcus aureus* (USA300). Overnight bacterial cultures were grown in optimal media for each species: Luria-Bertani (LB) broth for *E. coli*, *P. aeruginosa*, and *S. aureus*, and Todd-Hewitt (TH) broth for *S. pyogenes*. Cultures were pelleted and re-suspended in Mueller-Hinton (MH) broth for peptide screens or minimal inhibitory concentration (MIC) assays, with 1 × 10^6^ CFU/mL bacteria added per well (Wiegand et al., 2008). MICs for each parent peptide were identified against each bacterial strain as previously described (Fields et al., 2018). Members of each peptide library were screened against each bacterial strain at half of the MIC of the corresponding parent peptide to identify library members with increased antimicrobial activity relative to the parent peptide. Libraries whose parent peptides exhibited MICs >128 μM against a particular bacterial strain were not screened against that strain. Following each screen, peptides that exhibited antimicrobial activity were subjected to full MIC assays, as previously described (Fields et al., 2018). Minimal bactericidal concentrations (MBCs) were determined following the respective MIC assays by plating aliquots from wells corresponding to each peptide concentration from the 96-well plate used for the MIC on species-appropriate agar. Plates were incubated at 37°C overnight, and MBCs were identified as the lowest concentrations of peptide at which no colonies were obtained. A Synergy H1 Microplate Reader (BioTek, Winooski, VT, United States) was used to determine the MICs and identify peptides with greater antimicrobial activity than the corresponding parent peptide by measuring OD 600 of the bacterial cultures in 96-well microtiter plates.

### Peptide Cytotoxicity and Hemolysis Assays

To assess cytotoxicity of optimized peptides in mammalian cells, an immortalized human keratinocyte cell line (HaCaT) was used. Cells were grown to 80% confluency in 24-well culture dishes and washed with PBS (Thermo Fisher). Each peptide was diluted in fresh Dulbecco’s Modified Eagle Medium (DMEM), supplemented with 10% fetal bovine serum (FBS), to desired concentrations corresponding to MIC experiments and incubated with cells for 16 h in triplicate at 37°C, 5% CO_2_. After incubation, the cells were washed with sterile PBS, followed by a covered incubation with 4 μM ethidium homodimer (Molecular Probes) for 30 min at room temperature. Fluorescence was measured using the Synergy H1 Microplate Reader set to 528 nm excitation, 617 nm emission, and a cutoff value of 590 nm. Saponin (0.1% wt/vol, Sigma) was added to each well followed by a covered incubation for 20 min at room temperature, followed by another measurement on the Synergy H1 Microplate Reader using the same settings to normalize readings to the number of cells per well and determine the percent cytotoxicity. Peptides were characterized as cytotoxic at a particular concentration if incubation with that concentration of peptide resulted in more than 20% cell death after 16 h, which corresponded to the percent cytotoxicity in HaCaTs treated with the vehicle control under the same conditions.

To determine if peptide variants were hemolytic, fresh defibrinated whole sheep blood (Hardy Diagnostics) was washed three times in cold PBS, and washed blood was diluted 1:25 in PBS. Treatments were applied at a 1:10 ratio of sample:blood, where the sample was either 10% Triton, PBS, or a specific peptide concentration corresponding to concentrations used in the MIC assays. Treatments were incubated with the blood for 1 h at 37°C. Following incubation, samples were centrifuged at 0.4 × *g* for 10 min at 4°C, and the absorbance of the supernatants at 450 nm was measured on the Synergy H1 Microplate Reader to measure the release of hemoglobin. Percent hemolysis was quantified by normalizing to the negative and positive controls.

### Circular Dichroism Spectroscopy

Using a Jasco CD Spectrometer, peptide variants were scanned from 190 to 250 nm at 20 nm/min in a 2 mm cuvette. Peptides were diluted to a final concentration of 25 μM and suspended in either nanopure water, 9 mM SDS (Sigma-Aldrich), or 50% trifluoroethanol (TFE) (Sigma-Aldrich). Scans were run in triplicate and averaged. Blanks using the solvents were quantified to subtract from the peptide scans.

### Fluorescence Microscopy and Fluorescence-Activated Cell Sorting

To observe bacterial cell death in the presence of peptide in real time, fluorescence microscopy was employed using GFP-expressing *E. coli* strain BL21. MIC assays were conducted against BL21-GFP to determine the peptide concentration used for microscopy experiments. The peptide was suspended in PBS and added to diluted bacterial cells. GFP-expressing *E. coli* in the presence of peptide were imaged on the Eclipse Ti-E inverted microscope (Nikon) using GFP and DIC channels for 8 h at 37°C, with images taken every 10 min (Fields et al., 2020). Syn-xiamencin-2, a peptide with no detectable antimicrobial activity below 128 μM was also evaluated as a negative peptide candidate control.

For determination of bacterial cell death via membrane disruption, bacterial cells were subjected to propidium iodide (PI) uptake and quantified using fluorescence-activated cell sorting (FACS). *E. coli* strain BL21 were first diluted to a OD 600 of 1.0. Cells were washed three times in 0.85% NaCl (saline) solution and resuspended in either 1.5 mL of either saline, 70% isopropyl alcohol, or peptide diluted to either 2, 4, 8, 16 or 32 μM in saline for 1 h at ambient temperature. Following incubation, cells were pelleted and resuspended in 1 mL of saline with 1.5 μL/mL propidium iodide and incubated in the dark for 15 min. The samples were washed again with saline three times and run through a Beckman Coulter FC500 flow cytometer. The FL3 detector, using the propidium iodine PE-Alexa Fluor 610 emission filter, was used to sort the cells. 1 × 10^4^ events were collected for each sample run.

## Results

### Peptide Library Design

Previous studies demonstrated that the antimicrobial activity of full-length enterocin AS-48 can largely be attributed to cationic helices four and five (Montalbán-López et al., 2008). Since linearized forms containing this region have been shown to be active and capable of optimization, we hypothesized that homologous regions in similar enterocin AS-48-like natural sequences could also serve as a scaffold for peptide sequence optimization. We utilized the truncated 25 amino acid sequence from the active domain of enterocin AS-48 to bioinformatically search for other AS-48 like homologs having high conservation in this active domain, as was previously done to identify safencin AS-48 (Fields et al., 2018, 2020). Three additional species were identified with highly conserved AS-48-like domain regions, namely, *C. sordellii*, *P. larvae*, and *B. xiamenensis* (Supplementary Table S1). Each 25 amino acid sequence of the AS-48 domain was designated as the parent peptide (Peptide No. 1 in series) and used as a scaffold to build a series of library peptide variants with systematic amino acid substitutions to improve the activity of the original peptide. To increase affinity of the peptide variants to the anionic bacterial membrane, lysine was incorporated into the peptide, being substituted for a short-chained amino acid. In order to potentially increase penetration of the bacterial membrane, aliphatic and non-polar short-chained amino acids were substituted with the inclusion of the aromatic amino acid tryptophan, along with increasing the amphipathic nature of the peptides by flipping specific amino acids within the helical wheel. All changes were made in a step-wise fashion to build the peptide libraries. The four libraries, syn(synthetic)-enterocin, syn-sordellicin, syn-larvacin, and syn-xiamencin, were synthesized to 95% purity (GenScript).

### Screening and MIC Determination

Each parent peptide was first tested for MIC values against *E. coli* (BL21), *S. pyogenes* (M1), *P. aeruginosa* (PAO1), and *S. aureus* (USA300). All 95 peptide variants in each library were screened at one-half the MIC value of the parent peptide. Peptide variants within each library screen were determined to be a positive hit if inhibition was confirmed during screening. Further, if a peptide variant outperformed its parent scaffold against all screened bacterial species, the peptide was characterized as an optimized peptide variant and MIC assays were subsequently performed using these peptides. All parent peptide scaffolds were found to have a MIC above 128 μM against *S. aureus* USA300. Additionally, the parent peptide syn-larvacin-1 and syn-xiamencin-1 had MIC above 128 μM against PAO1.

### Syn-sordellicin Library

**FIGURE 1:**
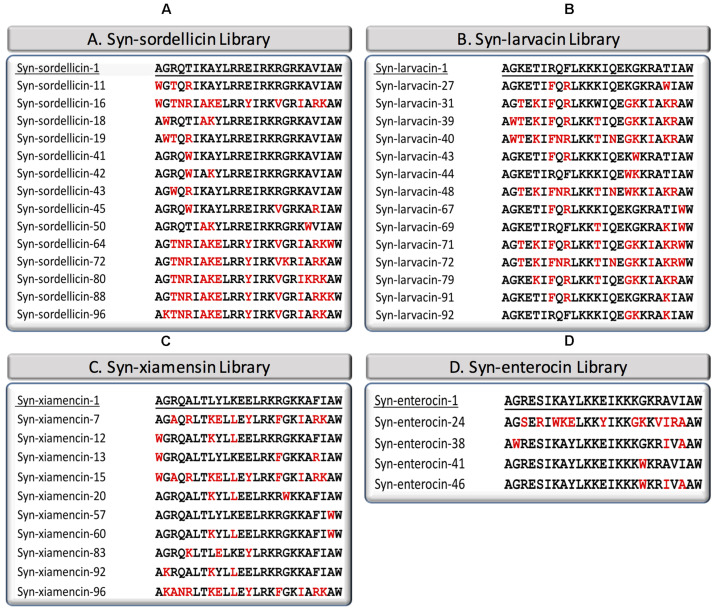
Amino acid sequence alignment of optimized variants from four AS-48 domain derived scaffold libraries. Syn-sordellicin library **(A)**, syn-larvacin library **(B)**, syn-xiamencin library **(C)**, and syn-enterocin **(D)** library indicate amino acid changes of optimized peptide variants, as compared to the parent scaffold, in red.

Syn-sordellicin-1 was determined to have a MIC of 8 μM against BL21, 8 μM against M1 and 16 μM against PAO1, thus this library was screened against BL21 at 4 μM, M1 at 4 μM, and PAO1 at 8 μM. Fourteen of the 95 screened peptides in this library were determined to be significantly antimicrobial against all three screened bacterial species (Figure 1A). Notably, syn-sordellicin-64, -80, and -96 exhibited MICs as low as 250 nM against BL21. Syn-sordellicin-11 and -16 were determined to have MIC against M1 at 500 and 250 nM, respectively. Against PAO1, syn-sordellicin-43 exhibited an MIC of 2 μM, which was the lowest MIC observed across all optimized peptide variants. Additionally, nine of the fourteen optimized peptide variants exhibited MIC below 128 μM against USA300; the best peptide variant against USA300 being syn-sordellicin-64 with a MIC and MBC of 8 μM (Table 1A).

**TABLE 1:** Antimicrobial activity of AS-48 domain derived peptide libraries. Broth microdilutions of optimized peptide variants within the syn-sordellicin **(A)**, syn-larvacin **(B)**, syn-xiamencin **(C)**, and syn-enterocin **(D)** libraries were used to determine antimicrobial activity against isolates of *E. coli*, *S. pyogenes*, *S. aureus*, and *P. aeruginosa*. MIC and MBC were established after 16 h incubation. Assays were done in triplicate and growth inhibition was determined by measuring the optical density at 600 nm.

	*E. coli* (BL21)		*S. pyogenes* (M1)		*S. aureus* (USA300)		*P. aeruginosa* (PAO1)	
Peptide Variant	MIC	MBC	MIC	MBC	MIC	MBC	MIC	MBC
**A.**								
Syn-sordellicin-1	8 μM	8 μM	8 μM	8 μM	>128 μM	>128 μM	16 μM	16 μM
Syn-sordellicin-11	2 μM	2 μM	0.5 μM	0.5 μM	>128 μM	>128 μM	8 μM	8 μM
Syn-sordellicin-16	1 μM	1 μM	0.25 μM	0.5 μM	16 μM	32 μM	8 μM	8 μM
Syn-sordellicin-18	1 μM	1 μM	4 μM	4 μM	32 μM	32 μM	4 μM	8 μM
Syn-sordellicin-19	2 μM	2 μM	2 μM	2 μM	>128 μM	>128 μM	8 μM	16 μM
Syn-sordellicin-41	2 μM	2 μM	2 μM	4 μM	>128 μM	>128 μM	16 μM	16 μM
Syn-sordellicin-42	2 μM	2 μM	4 μM	4 μM	>128 μM	>128 μM	16 μM	16 μM
Syn-sordellicin-43	1 μM	1 μM	2 μM	4 μM	32 μM	32 μM	2 μM	4 μM
Syn-sordellicin-45	1 μM	1 μM	2 μM	2 μM	32 μM	32 μM	16 μM	32 μM
Syn-sordellicin-50	1 μM	1 μM	2 μM	2 μM	>128 μM	>128 μM	8 μM	16 μM
Syn-sordellicin-64	0.25 μM	0.5 μM	1 μM	1 μM	8 μM	8 μM	8 μM	8 μM
Syn-sordellicin-72	0.5 μM	0.5 μM	1 μM	1 μM	16 μM	16 μM	16 μM	16 μM
Syn-sordellicin-80	0.25 μM	0.5 μM	2 μM	2 μM	64 μM	64 μM	16 μM	16 μM
Syn-sordellicin-88	1 μM	1 μM	1 μM	1 μM	64 μM	64 μM	16 μM	16 μM
Syn-sordellicin-96	0.25 μM	0.25 μM	0.5 μM	0.5 μM	32 μM	32 μM	8 μM	16 μM
**B.**								
Syn-larvacin-1	32 μM	>128 μM	8 μM	64 μM	>128 μM	>128 μM	>128 μM	>128 μM
Syn-larvacin-27	<0.0156 μM	<0.0156 μM	0.125 μM	0.5 μM	8 μM	8 μM	32 μM	64 μM
Syn-larvacin-31	1 μM	1 μM	0.25 μM	0.25 μM	8 μM	8 μM	64 μM	128 μM
Syn-larvacin-39	<0.0156 μM	<0.0156 μM	2 μM	4 μM	1 μM	4 μM	16 μM	64 μM
Syn-larvacin-40	2 μM	2 μM	0.25 μM	4 μM	4 μM	4 μM	16 μM	16 μM
Syn-larvacin-43	1 μM	1 μM	4 μM	4 μM	4 μM	4 μM	16 μM	16 μM
Syn-larvacin-44	0.5 μM	1 μM	0.5 μM	0.5 μM	32 μM	32 μM	16 μM	32 μM
Syn-larvacin-48	4 μM	4 μM	8 μM	8 μM	8 μM	8 μM	16 μM	32 μM
Syn-larvacin-67	4 μM	4 μM	0.25 μM	0.25 μM	64 μM	64 μM	16 μM	32 μM
Syn-larvacin-69	2 μM	16 μM	0.125 μM	0.5 μM	16 μM	16 μM	32 μM	32 μM
Syn-larvacin-71	0.25 μM	0.25 μM	0.125 μM	0.125 μM	<0.25 μM	<0.25 μM	16 μM	32 μM
Syn-larvacin-72	0.25 μM	0.25 μM	0.125 μM	2 μM	>128 μM	>128 μM	32 μM	64 μM
Syn-larvacin-79	16 μM	32 μM	>128 μM	>128 μM	4 μM	8 μM	64 μM	>128 μM
Syn-larvacin-91	1 μM	2 μM	0.5 μM	0.5 μM	2 μM	2 μM	32 μM	64 μM
Syn-larvacin-92	2 μM	2 μM	1 μM	1 μM	2 μM	8 μM	64 μM	128 μM
**C.**								
Syn-xiamencin-1	32 μM	32 μM	16 μM	16 μM	>128 μM	>128 μM	>128 μM	>128 μM
Syn-xiamencin-7	4 μM	4 μM	0.5 μM	0.5 μM	>128 μM	>128 μM	64 μM	128 μM
Syn-xiamencin-12	16 μM	16 μM	2 μM	2 μM	>128 μM	>128 μM	64 μM	64 μM
Syn-xiamencin-13	16 μM	32 μM	0.25 μM	0.25 μM	>128 μM	>128 μM	>128 μM	>128 μM
Syn-xiamencin-15	2 μM	2 μM	1 μM	1 μM	64 μM	128 μM	>128 μM	>128 μM
Syn-xiamencin-20	1 μM	1 μM	2 μM	2 μM	32 μM	32 μM	64 μM	64 μM
Syn-xiamencin-57	16 μM	16 μM	4 μM	8 μM	16 μM	32 μM	>128 μM	>128 μM
Syn-xiamencin-60	4 μM	8 μM	2 μM	4 μM	>128 μM	>128 μM	>128 μM	>128 μM
Syn-xiamencin-83	16 μM	32 μM	4 μM	4 μM	>128 μM	>128 μM	128 μM	>128 μM
Syn-xiamencin-92	4 μM	8 μM	4 μM	8 μM	8 μM	8 μM	16 μM	16 μM
Syn-xiamencin-96	2 μM	2 μM	4 μM	4 μM	8 μM	8 μM	16 μM	16 μM
**D.**								
Syn-enterocin-1	32 μM	32 μM	32 μM	32 μM	>128 μM	>128 μM	>128 μM	>128 μM
Syn-enterocin-24	2 μM	2 μM	16 μM	16 μM	64 μM	>128 μM	8 μM	16 μM
Syn-enterocin-38	4 μM	8 μM	16 μM	16 μM	>128 μM	>128 μM	32 μM	32 μM
Syn-enterocin-41	4 μM	4 μM	16 μM	16 μM	>128 μM	>128 μM	16 μM	16 μM
Syn-enterocin-46	2 μM	4 μM	8 μM	8 μM	32 μM	>128 μM	8 μM	16 μM

### Syn-larvacin Library

Syn-larvacin-1 was determined to have a MIC of 32 μM against BL21 and 8 μM against M1, therefore the peptide variants were screened at 16 and 4 μM, respectively. Fourteen of the 95 screened peptides outperformed its parent scaffold peptide against all four screened bacterial species (Figure 1B). Of these, five peptide variants exhibited MICs below 500 nM against BL21; syn-larvacin-27 and -39 exhibited MIC and MBC as low as 15.6 nM. Against M1, syn-larvacin-27, -69, -71, and -72 were confirmed to have MICs of 125 nM. As shown in Table 1B, the optimized peptides against USA300 showed a large range of activity, with MICs between 250 nM and 64 μM. Notably, syn-larvacin-71 had an MIC of 250 nM against USA300. Against PAO1, the fourteen syn-larvacin optimized peptides had MIC values between 16 and 64 μM.

### Syn-xiamencin Library

Syn-xiamencin-1 was determined to have a MIC of 32 μM against BL21 and 16 μM to M1, therefore the peptide variants of this library were screened at 16 μM against BL21 and 8 μM against M1. Ten optimized peptide variants were established (Figure 1C). The syn-xiamencin library was most effective against M1, establishing MICs to be as low as 250 and 500 nM for syn-xiamencin-13 and-7, respectively. Against BL21, many optimized peptide variants exhibited MICs and MBCs as low as 1 and 2 μM, including syn-enterocin-15, -20, and -96. Half of the optimized peptide variants against USA300 exhibited MICs below 128 μM, while eight of the ten exhibited MICs below 128 μM to PAO1. Between both bacterial species, successful peptide variants had MICs between 8 and 128 μM (Table 1C).

### Syn-enterocin Library

Syn-enterocin-1 was determined to have a MIC of 32 μM against BL21, 32 μM against M1, and 128 μM against PAO1, therefore all peptide variants were screened at 16 μM against BL21, 16 μM against M1 and 64 μM against PAO1. After screening, syn-enterocin-24, -38, -41, and -46 were confirmed to be optimized peptides for further studies (Figure 1D). These four peptide variants were then analyzed to confirm their individual MIC and MBC against the screened species, BL21, M1, and PAO1, along with assessing further activity against USA300 (Table 1D). It was determined that syn-enterocin-24 and -46 had MICs of 64 and 32 μM, respectively, against USA300, with MBC values above 128 μM.

### Cytotoxicity and Hemolytic Activity of Optimized Peptide Variants

Based on our previous screening and MIC determination, 42 optimized peptide variants with low MIC values against our panel of bacterial pathogens were selected for cytotoxicity assays against human keratinocytes (HaCaTs). HaCaT cytotoxicity was assessed by membrane permeabilization using ethidium homodimer assays. Four optimized peptide variants, all within the syn-sordellicin library, were found to be significantly cytotoxic (>30% cytotoxicity). Our most cytotoxic peptide was syn-sordellicin-64, exhibiting significant cytotoxicity in a dose-dependent manner, at 32 μM and above. Syn-sordellicin-19, -72, and -88 were also found to be cytotoxic at concentrations of 64 μM and above (Supplementary Figure S1). Due to their cytotoxicity, these four peptide variants were removed from further characteristic studies. All other optimized peptide variants, along with their parent peptide scaffold, were not cytotoxic at their established MIC concentrations. Across all peptide libraries, 29 of the optimized peptide variants exhibited minimal hemolytic activity (Supplementary Table S2). Of these, 17 showed hemolytic activity above 32 μM. Syn-sordellicin-64, in line with cytotoxicity data, exhibited hemolysis down to 250 nM. Syn-sordellicin-64 was found to be broadly antibacterial, including having the lowest MIC value against USA300 compared to the other syn-sordellicin peptides tested, giving reason to the cytotoxicity and hemolytic activity observed. Only peptide variants which showed no hemolytic activity below their MIC values were retained as potential antimicrobial candidates. These data suggest that our library approach to designing minimal peptides using AS-48 like bacteriocin domains results in very low eukaryotic toxicity overall, consistent with the natural forms of AS-48.

### Selection of Broad-Spectrum and Narrow-Spectrum Candidates

**FIGURE 2:**
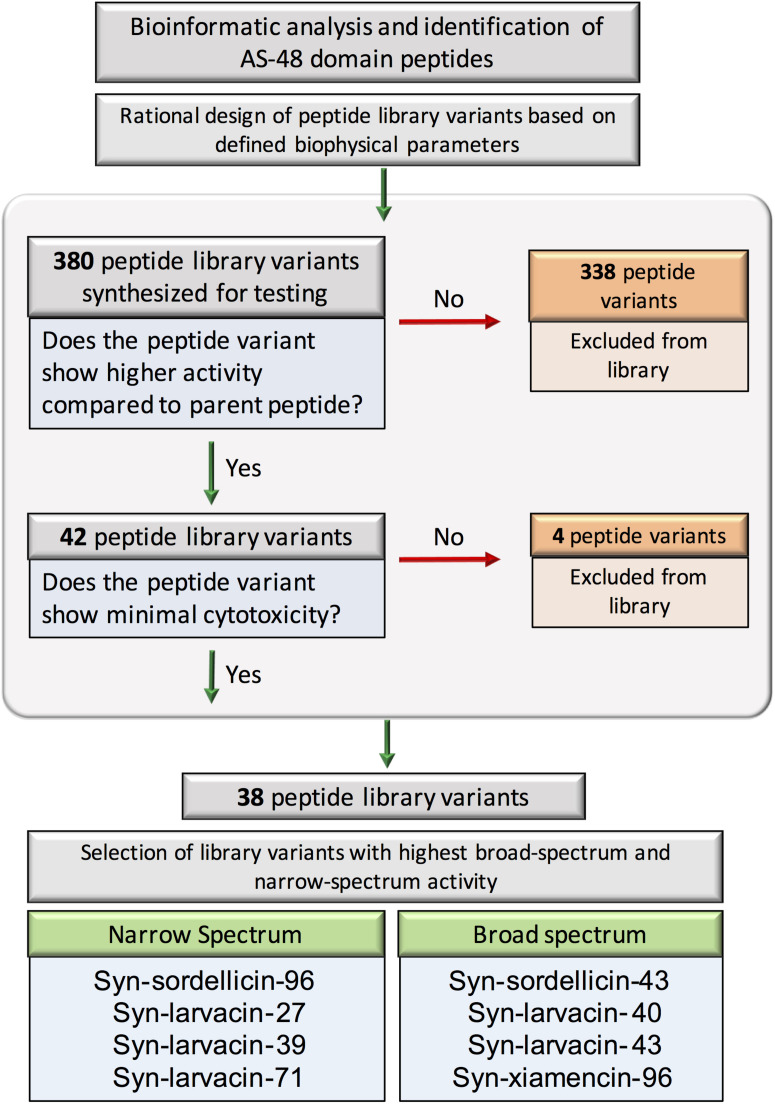
Flowchart showing the general strategy and summary for the design and evaluation of AS-48 domain- based minimal peptide libraries. Bioinformatic analysis and identification of AS-48 domain homologs resulted in 380 total peptide variants initially synthesized for testing. Peptides were excluded if they were outperformed by their parent scaffold in any of the species tested. Additional peptides with significant eukaryotic toxicity were also excluded. Eight peptides were identified to have the highest bactericidal activity either across all four tested species or the highest significant activity against single tested species; these were deemed the representative broad- and narrow-spectrum peptides, respectively and subject to further analysis.

We next categorized our optimized peptides into broad- and narrow-spectrum candidates based on their established MIC values, cytotoxicity and hemolytic activity (Figure 2). Optimized peptide variants were characterized as broad-spectrum candidates based on low MICs against a majority of the panel of bacterial pathogens tested. Based on these criteria, peptides were ranked and the top four, syn-sordellicin-43, syn-larvacin-40, syn-larvacin-43, and syn-xiamencin-96, were chosen as our broad-spectrum candidates to study further. Four peptides which exhibited narrow-spectrum bioactivity were selected as candidates. All narrow-spectrum peptide candidates had an established MIC below 250 nM to at least one of our tested bacterial species. Syn-sordellicin-96 and syn-larvacin-71 were found to have an MIC and MBC of 250 nM for BL21, while Syn-larvacin-27 and -39 had MIC and MBC below 15.6 nm. Against M1, syn-larvacin-27 and -71 had MICs of 125 nm. Syn-larvacin-71 also had the lowest MIC established against USA300 at 250 nM. The lowest MIC seen for PAO1 was syn-sordellicin-43 at 2 uM, a peptide chosen for broad-spectrum activity.

### Secondary Structure and Mode of Action Analysis of Optimized Peptide Variants

**FIGURE 3:**
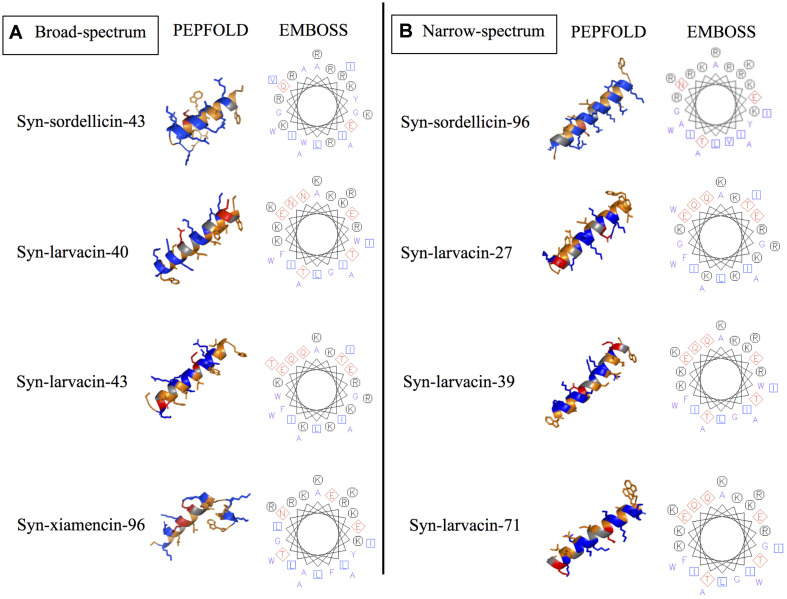
Structural prediction tools display residue substitutions of candidate peptide variants *in silico*. PEPFOLD was used for the prediction of the secondary structure of optimized peptide variants, while EMBOSS software pepwheel was used to visualize the location of residues looking down the helical axis. PyMOL was used to show hydrophobic (orange), cationic (blue), and anionic (red). These secondary structure and helical wheel projections show the chosen representatives for optimized peptide variants that showed broad-spectrum **(A)** and narrow-spectrum activities **(B)**.

**FIGURE 4:**
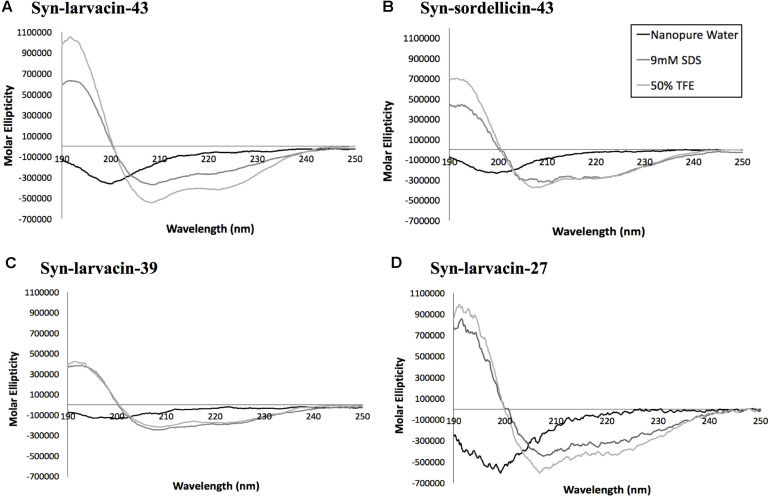
Assessment of secondary structures of candidate peptides by circular dichroism analysis. Broad-spectrum peptides, syn-larvacin-43 **(A)** and syn-sordellicin-43 **(B)**, and narrow-spectrum peptides, syn-larvacin-39 **(C)** and syn-larvacin-27 **(D)** were diluted to 25 μM in either nanopure water, 9 mM SDS or 50% TFE to observe folding behavior in these solvent systems. Spectral scans were collected in triplicate and averaged.

Secondary structures of each optimized peptide candidate were first analyzed using the prediction software PEPFOLD and visualized on PyMOL (Figure 3). All eight peptide candidates were shown to have alpha helical structures of various lengths. Visualization showed all analyzed peptide variants as amphipathic, having large hydrophobic and cationic regions on opposing sides of the helical structures. Helical wheel predictions using EMBOSS also showed a strong preference for amphipathicity (Figure 3). To validate our computational data, CD spectroscopy was done to observe the peptide structural behavior in controlled microenvironments. Using 9 mM SDS and 50% TFE to mimic the bacterial membrane environments, local minima were observed at 209 nm and 222 nm and a maximum peak at 192 nm in all four peptide variants, indicating the presence of an alpha-helical structure. In nanopure water, random coiling conformation is suggested by the observed dip around 195 nm (Figure 4). These data suggest that the peptides adopt an amphipathic alpha-helical structure with cationic regions, following the common theme for previously characterized AMPs (Wang et al., 2017; Fields et al., 2018).

**FIGURE 5:**
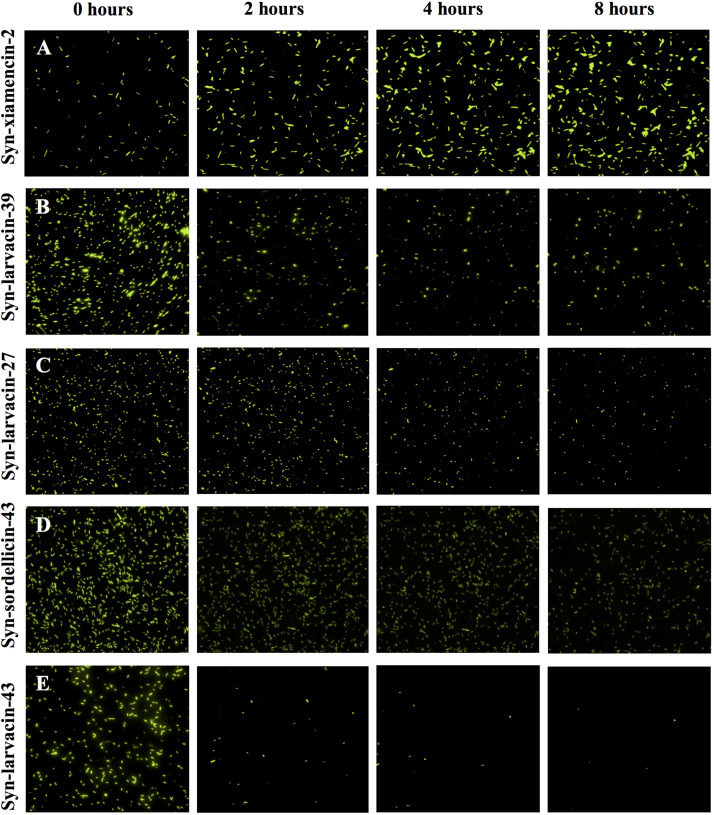
Live-imaging GFP-expressing *E. coli* incubated with optimized peptides. Cells were treated with 16 μM of each peptide variant and fluorescence was captured every 10 min for 8 h. 0, 2, 4, and 8 h time points show the change in fluorescence over time. Syn-xiamencin-2 **(A)** was used as a negative control, as it has no bactericidal activity against *E. coli*. Syn-larvacin-39 **(B)**, syn-larvacin-27 **(C)**, syn-sordellicin-43 **(D)**, and syn-larvacin-43 **(E)** each showed significant decrease in fluorescence over the 8 h, indicating GFP leakage.

**FIGURE 6:**
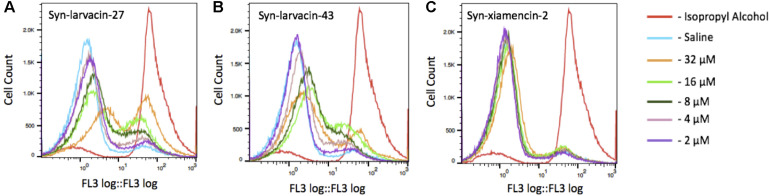
Fluorescence-activated cell sorting of propidium iodide stained *E. coli* cells. Bacterial cells were incubated for 1 h in the presence of isopropyl alcohol, saline or peptide variant. Isopropyl alcohol was used as a positive control, resulting in a large peak within the PI florescence range, indicated a large amount of cell death. While cells treated with syn-larvacin-27 **(A)** and syn-larvacin-43 **(B)** show higher cell counts of PI-stained cells in a dose-dependent manner. Treatment with syn-xiamencin-2 **(C)** was observed to have no significant difference with negative control cells treated with saline.

To assess bacterial membrane disruption as a potential mode of action for peptide candidates, we utilized live imaging of GFP-expressing *E. coli* BL21 and observed loss of membrane integrity in real-time over 8 h after incubation with peptides. As observed through live-imaging, GFP-expressing bacteria treated with Syn-xiamencin-2 showed increased fluorescence over the 8 h, indicating no GFP leakage (Figure 5A). However, GFP-expressing bacteria exhibited GFP leakage when treated with all tested optimized peptides, as shown by a decrease in fluorescence over the course of the 8 h (Figures 5B–E). GFP leakage in the presence of the optimized peptides suggests membrane disruption as the mode of action. In order to further examine if membrane disruption is the cause of bacterial cell death, permeabilization of the bacterial membrane by peptide candidates was assessed through a propidium iodide (PI) stain. If the bacterial membrane was disrupted, PI is able to enter cells and emit a fluorescent signal upon intercalation with DNA. Thus, fluorescence emitting cells are used as a proxy indicating cell death via membrane disruption. FACS analysis was used to sort PI positive cells in the presence of peptide candidates. Data collected showed an increase in PI positive cells, in a dose-dependent manner when incubated with syn-larvacin 27 and syn-larvacin 43 for 1 h, while syn-xiamencin-2 had no increase in PI positive cells, as compared to saline solution (Figure 6).

## Discussion

In this study, we demonstrate that the active helical domain of enterocin AS-48 and related homologs can be leveraged as basic scaffolds to build a substantial library of small, chemically synthesized AMPs that can be evaluated for activity. These peptide variants are designed using systematic biophysical parameters in order to obtain a large dataset. Larger datasets may give insight to how traits, such as hydrophobicity and amphipathicity, improve the activity and specificity of the bacteriocin variant from the original sequence. Importantly, we establish a general workflow that combines bioinformatic searches to discover novel bacteriocins to serve as initial peptide library scaffolds and biophysically guided rational design of synthetic AMP libraries.

Our results identify a large number of peptide variants possessing more potent bioactivity than their parent scaffold. Although in general terms, minimal domains of full-length bacteriocins are likely to display diminished antibacterial activity, our results provide evidence that the antimicrobial activity of truncated peptide candidates can be restored and even further improved using systematic amino acid substitution. Overall, our data support long-standing, general trends regarding AMP design. Namely, MICs can be decreased with amino acid substitutions that increase overall amphipathicity of the peptide. The replacement of short-chained amino acids, alanine and glycine, with a positively charged lysine, was also shown to increase potency of peptide variants. This is likely due to the ability to increase the cationic character of the peptide in key locations that served to increase the overall affinity of the peptide for the anionic bacterial membrane.

Another strategy we employed to increase the antimicrobial activity of the peptide variants was to replace aliphatic and non-polar short-chained amino acids with tryptophan, which increases the affinity of peptides to the interfacial regions of the bacterial membrane, aiding in peptide insertion. In some cases, substitution of glycine, a helix-breaking amino acid, for another amino acid, extends the helical nature of the peptide, which correlates to an overall increased helical propensity (Ong et al., 2014). This may be the cause of the observed increase in antimicrobial activity of some of our peptide variants. For example, replacement of glycine with either lysine or tryptophan, in the 2nd amino acid position on the N-terminus end was seen to improve antimicrobial activity as shown by syn-sordellicin-18, -16, -96, syn-larvacin-39, -40, syn-xiamencin-92, -96, and syn-enterocin-38. The location of the tryptophan substitution also correlated with lower MICs, with trends being observed within the peptide library groups. The syn-larvacin library showed increased antimicrobial activity with the substitution of a tryptophan in the 24th amino acid position, near the C-terminus. This was demonstrated in the cluster of improved antimicrobial activities observed between syn-larvacin-67 through syn-larvacin-72, which all contained tryptophan at the key amino acid position. In the syn-sordellicin and syn-xiamencin libraries, the substitution of tryptophan for the first N-terminal alanine contributed to five of our library candidates showing improved MICs.

A key method for increasing the amphipathic nature of peptide variants, hypothesized to increase peptide penetration into the hydrophobic core of the bacterial membrane, was to flip amino acids within the helical wheel (Wang et al., 2017). Many patterns emerge when further analyzing successful changes in amphipathicity of our optimized peptides. 18 of the 42 peptide variants that had flipped the positively charged amino acid in the 3rd position and the 5th position threonine, serine or alanine showed decreased MIC values. More specifically, our syn-sordellicin library successfully increased bioactivity of eight peptide variants through flipping of the 7th position lysine and 8th position alanine, six peptide variants through flipping the 9th position tyrosine and 13th position glutamic acid, and seven peptide variants through flipping the 16th position arginine and 21st position valine. Similar patterns were observed in the syn-larvacin library, with 11 peptide variants exhibiting increased antimicrobial activity from flipping the 7th position arginine and 9th position phenylalanine. Nine of these peptides were improved by flipping the 17th position lysine and 18th position glycine or tryptophan post substitution, and finally seven peptides showed increased bioactivity as a result of flipping the 19th position arginine and 23rd position isoleucine. These trends emerge in the other libraries as well, indicating the importance of changes in amphipathic nature of peptide scaffolds to increase antibacterial activity.

Despite the large number of library peptide candidates evaluated in this study, we observed remarkably low eukaryotic cytotoxicity rates from most of these peptides, consistent with the low overall observed eukaryotic toxicity inherent in full-length natural AS-48 bacteriocins. This demonstrates that despite the strong alpha-helical nature of the peptide variants, they do not exhibit significant membrane penetrating activity against eukaryotic cells. Significant host cell cytotoxicity was largely limited to four of the fifteen peptides in the syn-sordellicin library, with all other library variants showing appreciably no cytotoxicity to the HaCaT cell lines used in our assays. These data demonstrate that our minimal peptide libraries retain specificity to bacterial membranes, even with amino acid substitutions that improve activity against bacteria.

## Conclusion

In conclusion, our work demonstrates a general strategy of using minimal domains of natural bacteriocin sequences as scaffolds to design a substantial library of bacteriocin-based variants for evaluation. Given the general limitations in natural product isolation and purification, especially with regard to bacteriocins (Katz and Baltz, 2016), our approach allows for the use of chemical peptide synthesis to generate a large number of bacteriocin-based peptide variants for assessment. Further, the large number of bacteriocin variants that can be tested validate the biophysical approach to improve the activity of the peptide from which the natural product is based. Importantly, we show that genomics tools to discover related sequences of a known bacteriocin can be utilized as scaffolds to construct library variants that show improved activity over the original sequence. Future studies will be needed to determine the efficacy, stability, and toxicity of these peptide candidates *in vivo* and to evaluate their potential for use in clinical, agricultural and industrial settings.

## Data Availability Statement

All datasets presented in this study are included in the article/Supplementary Material.

## Author Contributions

JR, FF, and SL conceived of study and designed all experiments. VK, AG, SO’C, AZ, TM, DH, and KC assisted with experiments. JR and SL wrote the manuscript. KC, SL, JR, DH, and TM edited and assisted with the manuscript preparation including figure design. All authors contributed to the article and approved the submitted version.

## Conflict of Interest

The authors declare that the research was conducted in the absence of any commercial or financial relationships that could be construed as a potential conflict of interest.

## Supplementary Material

Supplementary Figure S1 Cytotoxic activity of four optimized peptides from the syn-sordellicin library. Peptide dilutions were added to HaCaT cell lines and incubated for 16 h. Cytotoxicity is classified as cell death occurring in >20% of cells.

Supplementary Table S1 BLASTp search of the alpha-helical domain of the AS-48 sequence from *E. faecalis* identified three species with homologous regions. The sequences are compared by showing fully conserved residues with (*). Residues which have similar biochemical properties are indicated by (:). When residues have opposing charges, they are shown with (.). Blank space shows no conservation of residues.

Supplementary Table S2 Hemolysis was evaluated using sheep erythrocytes. Incubation with each peptide was performed for 1 h and read at 450 nm. Percent hemolysis above 10% of positive control (Triton X-100) was characterized as significantly hemolytic in our evaluation.
