# Supplementary material for: Synthetic Peptide Libraries Designed From a Minimal Alpha-Helical Domain of AS-48-Bacteriocin Homologs Exhibit Potent Antibacterial Activity
Source: Front Microbiol. 2020 Nov 12;11:589666. doi: 10.3389/fmicb.2020.589666 (PMC7689250; doi:10.3389/fmicb.2020.589666)
Supplement: Supplementary Figure S1 — Cytotoxic activity of four optimized peptides from the syn-sordellicin library. Peptide dilutions were added to HaCaT cell lines and incubated for 16 h. Cytotoxicity is classified as cell death occurring in >20% of cells. [file Data_Sheet_1.pdf]

Supplementary table 1.

Library	Species	Parent Sequence
Syn-enterocin	*Enterococcus faecalis*	AGRESIKAYLKKEIKKKGKRAVIAW
Syn-sordellicin	*Clostridium sordellii*	AGRQTIKAYLRREIRKRGRKAVIAW
Syn-larvacin	*Paenibacillus larvae*	AGKETIRQFLKKKIQEKGKRATIAW
Syn-xiamencin	*Bacillus xiamenensis*	AGRQALTLYLKEELRKRGKKAFIAW
		`**::::  :*:.:::::*::* ***`

Supplementary figure 1.

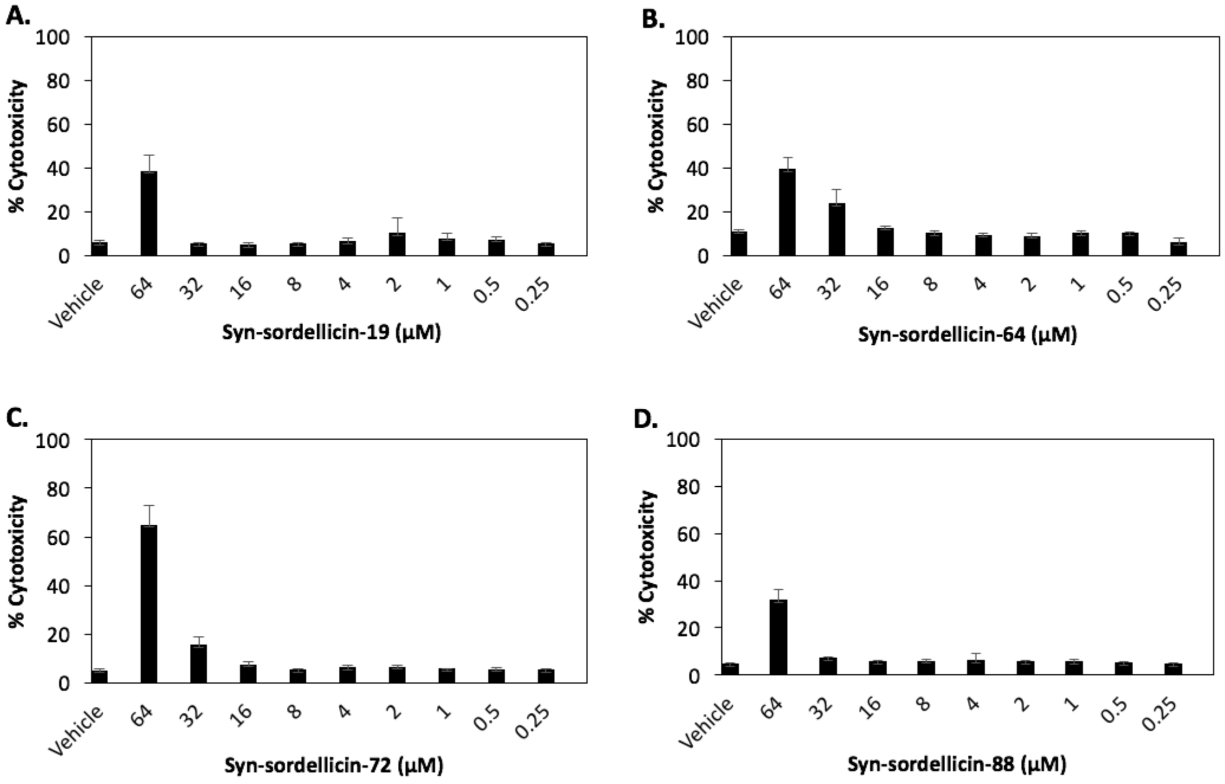

Supplementary table 2.

Peptide Variant	Hemolytic Concentration	Percent Hemolysis
Syn-sordellicin 11	>8 µM	21.3%
Syn-sordellicin 16	>32 µM	11.6%
Syn-sordellicin 18	>32 µM	14.1%
Syn-sordellicin 41	>32 µM	15.5%
Syn-sordellicin 42	>32 µM	11.9%
Syn-sordellicin 43	>8 µM	11.2%
Syn-sordellicin 50	>16 µM	11.5%
Syn-sordellicin 64	<0.25 µM	18.8%
Syn-sordellicin 80	>8 µM	11.3%
Syn-sordellicin 88	>2 µM	11.7%
Syn-sordellicin 96	>8 µM	14.6%
Syn-enterocin 24	>32 µM	10.5%
Syn-enterocin 38	>32 µM	14.4%
Syn-larvacin 39	>32 µM	14.4%
Syn-larvacin 40	>32 µM	17.5%
Syn-larvacin 43	>32 µM	19.9%
Syn-larvacin 44	>16 µM	20.9%
Syn-larvacin 67	>32 µM	16.3%
Syn-larvacin 69	>32 µM	12.5%
Syn-larvacin 71	>32 µM	20.6%
Syn-larvacin 72	>16 µM	26.3%
Syn-larvacin 91	>16 µM	23.4%
Syn-larvacin 92	>32 µM	29.9%
Syn-xiamencin 20	>16 µM	10.5%
Syn-xiamencin 57	>32 µM	17.0%
Syn-xiamencin 60	>32 µM	10.9%
Syn-xiamencin 83	>16 µM	10.7%
Syn-xiamencin 92	>32 µM	18.8%
Syn-xiamencin 96	>32 µM	20.0%
